# Rapid Diagnostic Stewardship and Blood Culture Use in a Pediatric Medical Center

**DOI:** 10.1001/jamanetworkopen.2025.35580

**Published:** 2025-10-06

**Authors:** Esther Vaugon, Cristina Costales, Zein Assad, Thomas Barter, Leila C. Posch, Etan Orgel, Deborah R. Liu, Jennifer Dien Bard

**Affiliations:** 1 Department of Pathology and Laboratory Medicine, Children’s Hospital Los Angeles, Los Angeles, California; 2 Department of Laboratory Medicine, Centre Hospitalier Universitaire Sainte-Justine, University of Montreal, Québec, Canada; 3 Keck School of Medicine, University of Southern California, Los Angeles; 4 Infection, Antimicrobials, Modelling, Evolution (IAME) Research Unit, INSERM UMR 1137, Paris Cité University, Paris, France; 5 Department of Pediatrics, Division of Infectious Diseases, Children’s Hospital Los Angeles, Los Angeles, California; 6 Department of Pediatrics, Division of Hematology-Oncology, Children’s Hospital Los Angeles, Los Angeles, California; 7 Department of Pediatrics, Division of Emergency Medicine, Children’s Hospital Los Angeles, Los Angeles, California

## Abstract

**Importance:**

A national shortage of blood culture bottles affected approximately half of US hospitals, necessitating changes in blood culture practices.

**Objective:**

To determine the association between restrictive blood culture stewardship measures and patient outcomes at a pediatric hospital.

**Design, Setting, and Participants:**

A retrospective cohort study was performed to determine the association between new restrictive stewardship measures and blood culture practices in a quaternary care pediatric medical center from July 1, 2023, to January 31, 2025. All children with blood cultures collected were included.

**Exposures:**

The following restrictive measures were enacted: (1) a 48-hour restriction on all aerobic blood cultures, (2) a 7-day restriction on all anaerobic blood cultures, (3) authorization to order an additional aerobic blood culture every 7 days, and (4) pooling of all central vein catheter lumens in 1 blood culture bottle.

**Main Outcomes and Measures:**

The primary outcome was the monthly blood culture positivity rate, stratified and normalized per 100 emergency department (ED) visits and per 100 patient-days.

**Results:**

During the study period, a total of 18 132 blood cultures from 5063 patient visits (median [IQR] age, 5.6 [1.1-12.4] years; 2744 [54.2%] male; 358 [7.15%] Black, 3013 [58.5%] Latino/a/x/Hispanic, and 720 [14.2%] White patients) were collected from August 1, 2023, to July 31, 2024 (preintervention period), and 6449 blood cultures from 2495 patient visits (1391 [55.8%] male, median [IQR] age; 5.5 [1.1-12.5] years; 191 [7.7%] Black, 1452 [58.2%] Latino/a/x/Hispanic, and 375 [15.0%] White patients) were collected from August 1, 2024, to January 31, 2025 (postintervention period). Restrictive stewardship measures were associated with a significant decrease in the blood culture collection rate per 100 ED visits (cumulative change, −24.1%; 95% CI, −38.4% to −8.9%; *P* = .01) and per 100 patient-days (cumulative change, −45.8%; 95% CI, −64.7% to −26.9%; *P* < .001). In the ED and inpatient units, the blood culture positivity rate remained stable, with a 14.4% (95% CI, −23.1% to 52.0%) and 27.8% (95% CI, −13.7% to 69.3%) cumulative increase, respectively. There was no significant change in hospital mortality due to septic shock (0.13% vs 0.15%) or mean length of stay (5.4 days vs 6.4 days), but there was a significant decrease in readmissions (3.3 vs 2.7; cumulative change, −27.5%; 95% CI, −7.6% to −47.6%; *P* = .02).

**Conclusions and Relevance:**

The implementation of restrictive blood culture measures, including pooling lumens and decreasing testing cadence, successfully reduced blood cultures collected without decreasing blood culture positivity rates and without an increase in mortality secondary to septic shock, readmission rates, or length of stay.

## Key Points

**Question:**

Can restrictive diagnostic stewardship measures effectively reduce blood culture usage in a pediatric population?

**Findings:**

In this cohort study with 24 581 blood cultures from 7558 patient visits, enforcement of restrictive stewardship measures was associated with a rapid reduction in blood culture utilization while maintaining stable blood culture positivity rates both in the emergency department and inpatient units. Hospital mortality due to septic shock, readmission rates, and length of stay did not increase after the intervention.

**Meaning:**

These findings suggest restrictive stewardship measures can effectively reduce blood culture utilization in pediatric settings, including among medically complex patients.

## Teaser

This cohort study evaluates the association between restrictive blood culture stewardship measures implemented at a pediatric hospital and patient outcomes.

## Introduction

Blood cultures are critical for diagnosing bloodstream infections (BSIs). In June 2024, BD Diagnostics, 1 of 3 major manufacturers of blood culture bottles in the US, alerted health care users of anticipated shortages due to supply chain disruptions.^1^ Following this, the US Food and Drug Administration issued a letter urging conservation strategies for blood culture use.^2^ The shortage impacted numerous hospitals, as approximately half of US laboratories use BD BACTEC systems.^3^ In February 2025, BD announced the global full recovery of its blood culture media, with optimal inventory levels restored after an 8-month shortage.^4^

To preserve the ability to perform blood cultures, health care systems had to implement contingency (<1 month’s supply) or crisis (<10 days’ supply) strategies.^3,5^ Balancing the urgent need to identify BSIs promptly with the necessity of conserving blood culture resources presented a significant challenge. This study aimed to evaluate the association between restrictive diagnostic stewardship measures and maintaining diagnostic performance for bacteremia in a clinical hospital setting while limiting adverse clinical outcomes. Our hypothesis was that the blood culture positivity rate would increase, as practitioners would reduce the number of low-yield blood cultures while still detecting true bacteremias.

## Methods

### Setting

Children’s Hospital of Los Angeles (CHLA) is a freestanding, quaternary care, pediatric medical center serving a diverse patient population. The hospital provides a full range of pediatric medical and surgical services. The CHLA institutional review board approved this study, which was conducted with a waiver of informed consent due to use of deidentified data. This study used Strengthening the Reporting of Observational Studies in Epidemiology (STROBE) reporting guidelines.

### Study Design and Population

We performed a single-center retrospective cohort study. All patients aged 21 years and younger with processed blood cultures from August 1, 2023, to January 31, 2025, were included. Blood cultures were processed as per standard protocol. We collected patients’ demographic data and detailed characteristics regarding the blood culture, including type of blood culture collected, blood volume collected, result, and time to positivity. All patients who had blood cultures collected during the 6-month study period were included. There was no predefined study size as inclusion was based solely on the occurrence of blood culture collection, which was determined by clinical indications and not under our control. As such, the final cohort size reflects the total number of patients meeting these inclusion criteria during the specified timeframe. Race was collected using the following self-reported categories: Asian, Black, Latino/a/x/Hispanic, White, other (includes American Indian, Alaska Native, Native Hawaiian, and Middle Eastern), and unknown/prefer not to say. Race was assessed in this study to assess whether diagnostic stewardship measures differentially affected access to testing across racial groups.

### Intervention: Restrictive Stewardship Measures and Education

To address the national blood culture bottle shortage, a multidisciplinary team was assembled, consisting of experts in clinical microbiology, infectious diseases, emergency medicine, hematology-oncology, health informatics, and supply chain. The team’s objective was to decrease blood culture use while minimizing potential risk to patients.

Before the shortage, blood cultures could be ordered without restrictions. To maintain adequate blood culture supplies, the following stewardship measures were implemented^1^: limiting the collection of aerobic blood cultures to once every 48 hours,^2^ limiting the collection of anaerobic blood cultures to once every 7 days,^3^ allowing 1 additional aerobic blood culture (aerobic #2) that could be ordered at any time at the physician’ discretion every 7 days,^4^ and pooling catheter lumens from the same central venous catheter (CVC) (eFigure 1 in Supplement 1).

The restrictive measures were integrated into the electronic medical record (EMR) system as a hard stop, but a bypass was allowed upon approval by the on-call microbiologist to support clinical judgment and ensure appropriate patient management. In the rare occasions where blood culture bottles were sent without EMR orders or microbiologist’s approval, those bottles were still incubated, and an educational phone call regarding appropriate processes was made to the clinician. Quality assurance data from CHLA supported limiting repeat daily blood cultures given the low diagnostic yield (eTable 1 in Supplement 1). Additionally, a guidance document (eFigure 2 in Supplement 1) was distributed to health care professionals outlining the clinical indications for blood culture collection based on evidence-based recommendations.

### Study Periods

We defined 2 periods according to the implementation of restrictive stewardship measures. The first was the preintervention period, from August 1, 2023, to July 31, 2024, and the second was the postintervention period, from August 1, 2024, to January 31, 2025.

### Outcome Measures

The primary outcome was the monthly blood culture positivity rate stratified and normalized per 100 emergency department (ED) visits and per 100 patient-days (ie, the proportion of blood cultures positive for pathogenic organisms relative to the total number of blood cultures performed). This rate excluded contaminants and blood cultures that tested positive for the same organism in the same patient within 7 days (repeat positive blood cultures). Blood culture positivity rate was chosen as an indicator of diagnostic performance. A sustained or increased positivity rate in the postintervention period would suggest improved diagnostic stewardship (ie, that lower-yield cultures were avoided while detecting true bacteremia).

Secondary outcomes were (1) the rate of blood cultures collected per 100 ED visits and per 100 patient-days, (2) the proportion of blood culture bottles with appropriate blood volume for age,^6^ (3) the mean length of hospital stay, (4) the readmission rates within 7 days of discharge, and (5) the mortality rates, including mortality rates due to suspected septic shock. All deaths from suspected septic shock were reviewed for possible delays in blood culture collection, noted either by the clinical team or identified during our review.

To assess mortality due to septic shock, we reviewed autopsy reports, death certificates, and discharge summaries to determine whether septic shock contributed to the patient’s death. For classification of bloodstream contaminant vs pathogen, we applied the College of American Pathologists (CAP) definition^7,8^ as well as reviewed all medical records where a single blood culture set was positive for organisms listed in the Centers for Disease Control and Prevention National Healthcare Safety Network common commensal list,^9^ categorizing them as contaminants or potential pathogens based on the documentation of the treating team (eFigure 3 in Supplement 1). Finally, *Streptococcus mitis/oralis* is part of the viridans group streptococci which is considered a contaminant as per the CAP definition. However, given the known pathogenicity of *Streptococcus mitis/oralis* in oncologic patients, medical record review was conducted for all single set positive *S. mitis/oralis* cultures to determine if the isolate was considered a pathogen or contaminant.

To minimize potential sources of bias, several steps were taken. First, inclusion criteria were based on routinely collected blood cultures, reducing the risk of selection bias. Second, all blood culture data during the study period were extracted from standardized orderables in the EMR to limit information bias.

### Statistical Analysis

The outcomes of the restrictive measures on the monthly blood culture collection, blood culture positivity rates, and balancing measures (readmission rates, length of stay, overall mortality, and mortality rates due to septic shock) were estimated by the means of a segmented linear regression model. The time unit was 1 month to provide a sufficient number of events per time unit and enough statistical power.^10^ We included a dummy variable in the model to estimate the changes in blood culture collection, positivity rates, and each balancing measure, assuming that the intervention would be associated with an immediate change in these outcomes. Based on data from the 12 preintervention months, the model was used to generate a counterfactual scenario assuming that the restrictive stewardship measures had not taken place. The estimated cumulative change was expressed as the percentage change between the rate fitted by the model and the estimated counterfactual rate, which was calculated for each time point of the intervention period. The validity of the segmented regression model was assessed by visual inspection of the correlograms (autocorrelation and partial autocorrelation functions) and residuals analysis. We verified whether the residuals of the models were normally distributed and showed constant variance over time.^11^

For the remaining secondary outcomes, continuous variables were compared using Wilcoxon rank-sum tests or *t* tests, and categorical variables were compared using χ^2^, as appropriate. A 2-sided *P* < .05 was considered statistically significant. All statistical analyses were performed using R version 4.3.3 (R Project for Statistical Computing).

## Results

### Patient Characteristics

During the study period, a total of 18 132 blood culturesfrom 5063 patient visits (2744 [54.2%] male, median [IQR] age; 5.6 [1.1-12.4] years; 358 [7.15%] Black, 3013 [58.5%] Latino/a/x/Hispanic, and 720 [14.2%] White patients) were collected from August 1, 2023, to July 31, 2024 (preintervention period), and 6449 blood cultures from 2495 patient visits (1391 [55.8%] male, median [IQR] age; 5.5 [1.1-12.5] years; 191 [7.7%] Black, 1452 [58.2%] Latino/a/x/Hispanic, and 375 [15.0%] White patients) were collected from August 1, 2024, to January 31, 2025 (postintervention period). Over the study period, the distribution of admission units among patients from whom blood culture bottles were collected shifted with a decrease in medical or surgical inpatient and outpatient units and an increase in ED, hematology-oncology, and intensive care units. Patient demographics are summarized in Table 1.

**Table 1.** Demographic Characteristics of Patients With Blood Cultures Collected Before and After Restrictive Stewardship Measures in a Pediatric Hospital

Demographics	Patients, No. (%)		*P* value^a^
	Preintervention (n = 5063 visits)	Postintervention (n = 2495 visits)	
Age, median (IQR), y	5.6 (1.1-12.4)	5.5 (1.1-12.5)	.67
Sex			
Female	2319 (45.8)	1104 (44.2)	.20
Male	2744 (54.2)	1391 (55.8)	
Race and ethnicity			
Asian	336 (6.6)	158 (6.3)	
Black	358 (7.1)	191 (7.7)	
Latino/a/x/Hispanic	3013 (59.5)	1452 (58.2)	<.001
White	720 (14.2)	375 (15.0)	
Other^b^	393 (7.8)	149 (6.0)	
Unknown/prefer not to say	241 (4.8)	170 (6.8)	
Hospital unit			
Medical/surgical	2069 (40.9)	907 (36.4)	<.001
Hematology-oncology	1037 (20.5)	567 (22.7)	
Emergency department	1027 (20.3)	526 (21.1)	
PICU	414 (8.2)	225 (9.0)	
Outpatient	321 (6.3)	129 (5.2)	
NICU	195 (3.9)	141 (5.7)	

Abbreviations: NICU, neonatal intensive care unit; PICU, pediatric intensive care unit.

^a^ Analysis by Wilcoxon rank sum test and Pearson χ^2^ test.

^b^ Other included American Indian, Alaska Native, Native Hawaiian, and Middle Eastern.

### Primary Outcome

Before our stewardship measures, the mean blood culture positivity rate estimated by the model was 2.1% (95% CI, 1.7% to 2.5%) per month in the ED. We observed a nonsignificant increase in the blood culture positivity rate postintervention (cumulative increase, 14.4%; 95% CI, −23.1% to 52.0%) (Table 2 and Figure). Overall, postintervention, the mean blood culture positivity rate per month estimated by the model was 2.7% (95% CI, 2.2% to 3.1%).

**Table 2.** Primary and Secondary Outcomes Before and After Implementation of Restrictive Stewardship Measures

Outcome	Mean monthly rate (95% CI)		Cumulative change over the period, % (95% CI)^a^	*P* value for cumulative change^b^
	Preintervention period	Postintervention period		
Primary outcome: monthly blood culture positivity, %				
ED	2.1 (1.7 to 2.5)	2.7 (2.2 to 3.1)	14.4 (−23.1 to 52.0)	.46
Inpatient	1.4 (1.1 to 1.7)	2.0 (1.7 to 2.3)	27.8 (−13.7 to 69.3)	.21
Secondary outcome: monthly blood culture collected				
ED (per 100 ED visits)	6.4 (5.7 to 7.3)	5.7 (4.8 to 6.6)	−24.1 (−8.9 to −38.4)	.007
Inpatient (per 100 patient-days)	9.2 (8.3 to 10.1)	5.2 (4.2 to 6.2)	−45.8 (−26.9 to −64.7)	<.001
Secondary outcomes: balancing measures				
7-d readmission, %	3.3 (3.0 to 3.6)	2.7 (2.3 to 3.0)	−27.5 (−7.6 to −47.6)	.017
Mean length of stay, d	5.4 (4.9 to 6.1)	6.4 (5.7 to 7.3)	−6.8 (−19.1 to 5.6)	.29
Mortality, all causes, %	0.50 (0.40 to 0.68)	0.68 (0.55 to 0.81)	72.4 (6.3 to 138.5)	.05
Mortality from suspected septic shock, %	0.13 (0.07 to 0.16)	0.15 (0.09 to 0.21)	−8.3 (−82.7 to 66.2)	.83

^a^ Mean percentage change between the rate fitted by the model and the estimated counterfactual rate; calculated for each time point of the intervention period.

^b^ Analysis by segmented linear regression.

**Figure.**
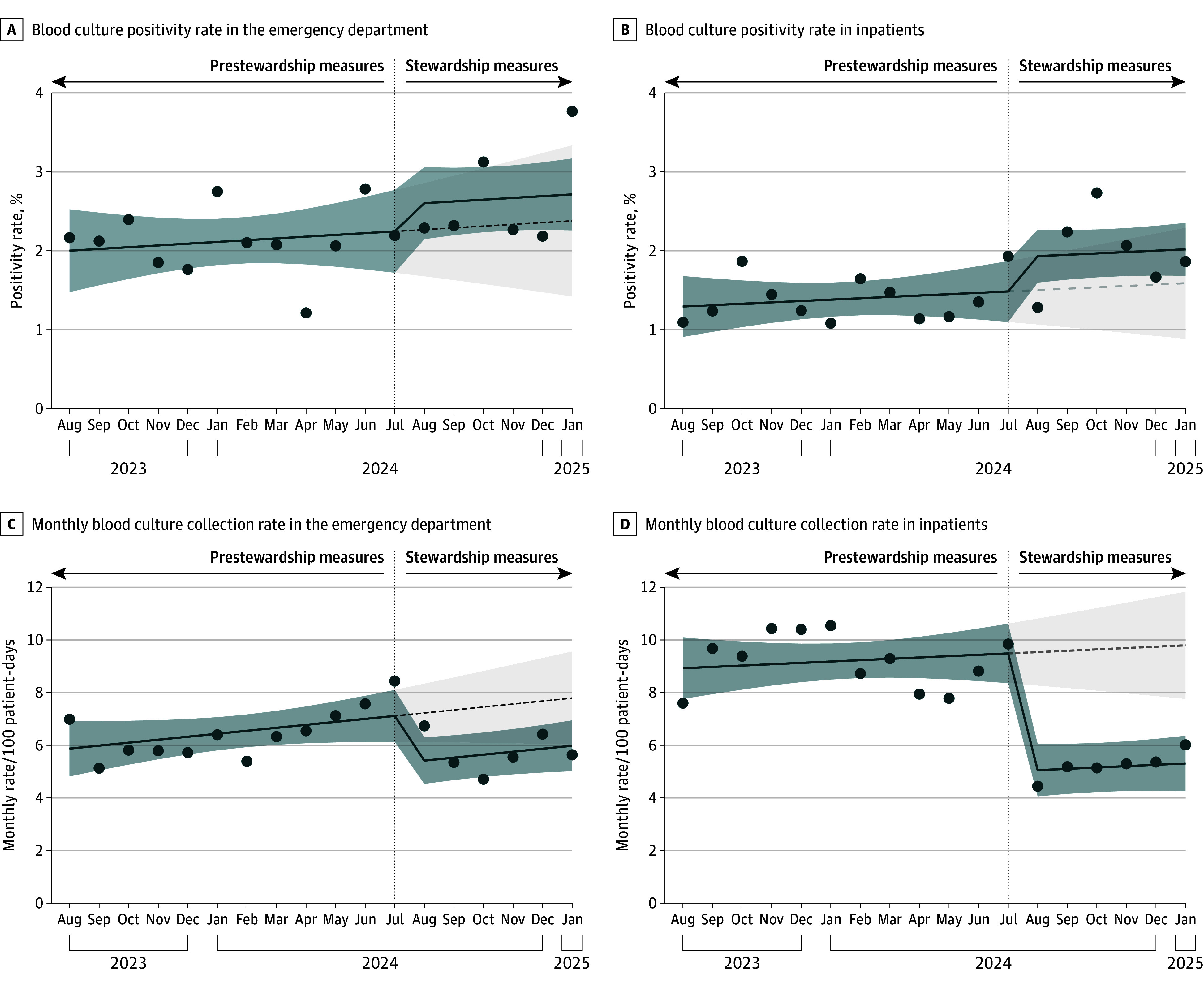
Blood Culture Collection Rate and Positivity Rate in the Emergency Department and Inpatient Units Blood culture positivity rate excluding contaminants and blood cultures that tested positive for the same organism in the same patient within 7 days (repeat positive blood cultures). Solid line indicates the segmented linear regression model estimate; dashed line indicates the counterfactual scenario without diagnostic stewardship measures; dots indicate the observed data; dark shaded area indicates the 95% CI for the model estimate; and light shaded area indicates the 95% CI for the counterfactual trend.

In the inpatient units, before our stewardship measures, the mean blood culture positivity rate per month estimated by the model was 1.4% (95% CI, 1.1% to 1.7%). We observed a nonsignificant increase in the blood culture positivity rate postintervention (cumulative change, 27.8%; 95% CI, −13.7% to 69.3%) (Table 2 and Figure). Postintervention, the mean blood culture positivity rate in the inpatient units estimated by the model was 2.0% (95% CI, −2.3% to 1.7%). The quality assessment of the model was satisfactory (eFigure 5 in Supplement 1).

### Secondary Outcomes

In the ED, restrictive stewardship measures were associated with a significant decrease in the monthly rate of blood culture bottles processed per 100 ED visits (cumulative decrease, 24.1%; 95% CI, −38.4% to −8.9%, *P* = .01) (Table 2 and Figure). An even greater decrease in the monthly rate of blood culture bottles processed per 100 patient-days was observed in the inpatient units (cumulative decrease, 45.8%; 95% CI, −64.7% to −26.9%; *P* < .001) (Table 2 and Figure). Over the 6-month intervention period, we estimate that 632 (95% CI, –248 to 2036) blood cultures were averted in the ED and 2800 (95% CI, 1167 to 4434) in the inpatient service. Notably, the overall reduction in blood culture collection was primarily due to a 52.2% decrease in repeat blood cultures, which was defined as cultures collected more than 1 hour after the initial sample during the same patient visit (Table 3).

**Table 3.** Blood Culture (BC) Characteristics Before and After Restrictive Stewardship Measures

Characteristic	Median (IQR)		*P* value
	Preintervention	Postintervention	
BC collection			
Total No.	18 132	6449	NA
BC collected per day	49 (42-57)	32 (28-41)	<.001
Repeat BC collected per day^a^	23 (17-28)	11 (8-14)	<.001
Positive BC, No. (%)	1109 (6.1)	437 (6.8)	.07
Pathogens (first positive BC)	309 (1.7)	150 (2.3)	<.001
Pathogens (additional positive BC)	537 (3.0)	150 (2.3)	.009
Time to positivity, h	18.0 (12.0-25.0)	15.0 (10.5-23.0)	.001
BC volume			
Total No.	17 652^b^	6285^b^	NA
Total BC volume collected per day	144.1 (115.8-169.3)	106.0 (83.6-125.1)	<.001
BC volume, mL	2.2 (1.3-4.1)	2.2 (1.3-4.2)	.81
Pathogens	2.7 (1.6-4.4)	3.0 (1.6-5.1)	.03
Contaminants	1.5 (0.7-2.3)	1.6 (0.7-2.3)	.66
Negative	2.2 (1.3-4.1)	2.2 (1.2-4.2)	.79
Appropriate volume for age, No. (%)	4795 (27.2)	1880 (29.9)	<.001
Inpatient	3695 (33.9)	1397 (43.0)	<.001
Emergency department	861 (14.1)	400 (14.3)	.79
Outpatient	239 (38.4)	83 (34.9)	.38

Abbreviation: NA, not applicable.

^a^ Repeat BC: BC repeated for an identical patient visit more than 1 hour after the original collection.

^b^ Blood volume recorded for 17 652 of 18 132 (97.4%) and 6285 of 6449 (97.5%) BC bottles in each respective intervention period.

### Balancing Measures

Balancing measures during the preintervention and postintervention periods are summarized in Table 2 and eFigure 4 in Supplement 1. Neither the overall mortality rate (0.50% to 0.69% of all admissions) nor mortality in patients with suspected septic shock (0.13% to 0.15%) changed significantly. Hospital readmission rates within 7 days decreased between the 2 study periods while mean length of stay remained stable (5.4 days vs 6.4 days).

To ensure blood cultures were collected at the appropriate time, we reviewed 25 deaths from suspected septic shock in the preintervention period and 15 in the postintervention period. Only 1 case in the preintervention period involved a possible delay in blood culture collection. In the postintervention period, no delays in blood culture collection were identified. No safety reports related to blood culture testing were submitted to the quality department.

### Blood Culture Characteristics

During the study period, we observed a 26.4% reduction in the total blood volume received at our laboratory for culture (from a mean [SD] of 144.1 [40.3] to 106.0 [32.3] mL; mean difference, 38.1; 95% CI, 31.9-44.4; *P* < .001). Additionally, the proportion of blood cultures with volumes appropriate for patient age slightly increased from 27.2% to 29.9% (χ^2^_1_ = 17; *P* < .001) (Table 3). Furthermore, the median (IQR) time to positivity slightly decreased from 18 (12-25) to 15 (10.5-23) hours (median difference estimate, 1.5; 95% CI, 0.5-2.7; *P* < .001). The median volume for blood cultures positive for pathogens also increased from 2.7 (1.6-4.4) mL to 3.0 (1.6-5.1) mL (median difference estimate, 0.3; 95% CI, 0.01-0.5; *P* = .03) (Table 3). Finally, pooling lumens did not appear to increase rates of contamination (0.2% for pooled vs 0.8% for single) (eTable 2 in Supplement 1).

## Discussion

In response to a blood culture bottle shortage, restrictive measures on blood culture ordering were implemented within our EMR. This intervention led to a cumulative reduction of blood cultures collected by 24.1% in the ED and 45.8% in inpatient services. While a reduction was expected given the restrictive nature of the intervention, our primary interest was whether this reduction would significantly change diagnostic yield. Interestingly, we observed a slight, albeit statistically nonsignificant, increase in blood culture positivity rates in both ED and inpatient settings. One possible explanation for this slight increase is a realignment toward prioritizing the patients with the most severe illnesses, as reflected in a shift in admission patterns: more blood cultures were collected from patients admitted to the ED, intensive care, and hematology-oncology units than from those in medical-surgical units or outpatient settings. Importantly, mean length of stay, readmission rates or mortality due to suspected septic shock did not change significantly.

Our findings are consistent with other studies demonstrating that restrictive diagnostic stewardship measures can reduce blood culture usage by 20% to 49% during a resource shortage^12,13,14,15^ without negatively impacting the overall blood culture positivity rate.^14,15^ These interventions enabled us to maintain our blood culture bottle stock without resorting to crisis management measures, such as using expired blood culture bottles or forgoing blood culture testing altogether.^3^

Furthermore, our stewardship intervention led to a 26.4% reduction in the total blood volume received daily by the laboratory for blood cultures. Phlebotomy-related blood loss is an increasingly recognized concern, particularly among neonatal ICU patients, who may lose between 10% and 90% of their total blood volume to phlebotomy within the first 2 weeks of life.^16^ By reducing unnecessary blood culture collection, such stewardship efforts may offer the added benefit of minimizing blood loss from sampling.

Our stewardship measures also included pooling catheter lumens from the same CVC. We observed a slight increase in the recovery of pathogens without an increase in contamination rates. The practice of pooling CVC cultures remains contentious in the literature. While 1 study^17^ demonstrated comparable sensitivity between pooled and individual-lumen CVC cultures, another study^18^ reported lower sensitivity with pooling. Unfortunately, prospective studies specifically evaluating the diagnostic accuracy of pooling CVC in pediatric populations are still lacking.

Another key aspect of our intervention was the reduction in the number of repeat blood cultures, with a 52.1% decrease in the median number of repeat orders. This decreased the number of additional blood cultures positive for the same pathogen without appearing to significantly impact patient outcomes. The role of repeat blood cultures in BSI remains debated.^19,20,21,22^ A recent pediatric study found that *Staphylococcus aureus* and yeast species were associated with persistent BSIs, while anaerobes and *Streptococcus* species were never recovered from repeat cultures.^23^ This evidence may inform future diagnostic stewardship guidelines, allowing for exceptions for repeat blood cultures within 48 hours when prior cultures were positive for *Staphylococcus aureus* or yeast species.^12^

While contingency and crisis strategies are necessary during a shortage, they are often deimplemented when supply levels stabilize.^3^ The blood culture bottle shortage has provided a unique opportunity for us to reevaluate our pediatric center’s blood culture practices. Encouraged by the lack of safety signals emerging from our data, our pediatric quaternary care center decided to permanently enact these restrictive measures as of April 24, 2025. Given the most frequent reason for additional blood culture bottles was documentation of pathogen clearance (especially for *Staphylococcus aureus* and *Candida* species), we coordinated with our informatics department to lift all EMR restrictions when a patient had a documented positive blood culture in the last 7 days.

The shortage also allowed us to identify potential areas of improvement in our blood culture practices. Given the subpar proportion of blood culture bottles with appropriate blood volume before and after the intervention (<30%), we are now focusing on improving blood culture fill volumes in the context of a longitudinal quality improvement plan.

Finally, we would like to acknowledge that these stewardship practices were only possible because of the workforce available. The microbiology on-call team is composed of 2 microbiologists and 2 fellows that are available around the clock to promptly support clinical teams, such as in cases of suspected endocarditis where 3 blood cultures obtained from separate venipunctures are recommended.^24^

### Limitations

This study had several limitations. First, as a single-center study, our findings may not be fully generalizable to other settings. Second, the postintervention period was only 6 months, which limited our ability to be adequately powered to detect rare outcomes such as death due to septic shock. Seasonal shifts in illnesses in the postintervention period were also not fully captured as our postintervention period was less than 1 year. Third, we did not assess other potential balancing measures such as delays of central line removals or delays in blood culture collection across all visits. Fourth, our definitions of contaminants and suspected septic shock may have led to some misclassification of outcomes. In pediatric populations, it is not always feasible to obtain 2 separate blood culture sets (the reference standard for distinguishing pathogens from contaminants), and adjudicating death due to septic shock was challenging, as autopsy reports were unavailable for the majority of cases. However, these potential misclassifications are likely to have been consistent across both study periods and are unlikely to have biased the results.

## Conclusions

The implementation of restrictive blood culture measures, including pooling lumens and restricting testing cadence, successfully reduced blood culture utilization during a nationwide blood culture shortage. These interventions did not significantly change overall blood culture positivity, hospital length of stay, readmission rates or mortality due to suspected septic shock in our 6-month follow-up period. Additional studies with prolonged follow-up periods are needed to assess potential unintended consequences as diagnostic stewardship measures are implemented in pediatric populations.

## Notes

Supplement 1. eTable 1. Diagnostic Yield of Repeated Blood Cultures in Preintervention Period (August 2023 to July 2024) eTable 2. Distribution of Blood Culture Results According to Fill Volumes and Type of Draw During the Entire Study Period (For Both Pre- and Postintervention Periods) eFigure 1. Restrictive Blood Culture Measures Enacted Via Electronic Medical Records eFigure 2. Syndromic Guidance for Appropriate Blood Culture Use eFigure 3. Classification of Contaminants vs Pathogens eFigure 4. Readmissions Rates, Mean Length of Stay, Mortality and Mortality Secondary to Suspected Septic Shock in the Pre- and Postintervention Period eFigure 5. Correlograms and Residuals Analysis

Supplement 2. Data Sharing Statement
