# Supplementary material for: Rapid Diagnostic Stewardship and Blood Culture Use in a Pediatric Medical Center
Source: JAMA Netw Open. 2025 Oct 6;8(10):e2535580. doi: 10.1001/jamanetworkopen.2025.35580 (PMC12501814; doi:10.1001/jamanetworkopen.2025.35580)
Supplement: Supplement 1. — eTable 1. Diagnostic Yield of Repeated Blood Cultures in Preintervention Period (August 2023 to July 2024) eTable 2. Distribution of Blood Culture Results According to Fill Volumes and Type of Draw During the Entire Study Period (For Both Pre- and Postintervention Periods) eFigure 1. Restrictive Blood Culture Measures Enacted Via Electronic Medical Records eFigure 2. Syndromic Guidance for Appropriate Blood Culture Use eFigure 3. Classification of Contaminants vs Pathogens eFigure 4. Readmissions Rates, Mean Length of Stay, Mortality and Mortality Secondary to Suspected Septic Shock in the Pre- and Postintervention Period eFigure 5. Correlograms and Residuals Analysis [file jamanetwopen-e2535580-s001.pdf]

Supplemental Online Content

Vaugon E, Costales C, Assad Z, et al. Rapid diagnostic stewardship and blood culture use in a pediatric medical center. *JAMA Netw Open*. 2025;8(10):e2535580. doi:10.1001/jamanetworkopen.2025.35580

eTable 1. Diagnostic Yield of Repeated Blood Cultures in Preintervention Period (August 2023 to July 2024)

eTable 2. Distribution of Blood Culture Results According to Fill Volumes and Type of Draw During the Entire Study Period (For Both Pre- and Postintervention Periods)

eFigure 1. Restrictive Blood Culture Measures Enacted Via Electronic Medical Records

eFigure 2. Syndromic Guidance for Appropriate Blood Culture Use

eFigure 3. Classification of Contaminants vs Pathogens

eFigure 4. Readmissions Rates, Mean Length of Stay, Mortality and Mortality Secondary to Suspected Septic Shock in the Pre- and Postintervention Period

eFigure 5. Correlograms and Residuals Analysis

This supplemental material has been provided by the authors to give readers additional information about their work.

© 2025 Vaugon E et al. *JAMA Network Open*.

eTable 1. Diagnostic yield of repeated blood cultures* in preintervention period (August 2023 to July 2024)

	No. (%)	
	**AEROBIC** (n = 11,983)	**ANAEROBIC** (n = 6,049)
Total repeated[1] BC	875 (7.3)	475 (7.9)
**Repeat Blood Culture Characteristics**	**AEROBIC** (n = 875)	**ANAEROBIC** (n = 475)
Positive BC after previously negative BC	41 (4.7)	3 (0.6)
Positive BC identified "repeat"[2] pathogen	14 (1.6)	0 (0)
First occurrence of a contaminant	16 (1.8)	0 (0)
First occurrence of a pathogen	11 (1.3)	3 (0.6)
First pathogen (isolated)	8 (0.9)	0 (0)
First pathogen (additional BC positive)	3 (0.3)	3 (0.6)

BC: Blood cultures
[1]Repeated within 48 hours for aerobic BC and within 7 days for anaerobic BC for the same visit
[2]"Repeat" pathogen previously isolated from an aerobic blood culture collected >48 hours but <7 days earlier

eTable 2. Distribution of blood culture results according to fill volumes and type of draw during the entire study period (for both pre- and postintervention periods)

	No. (%)			
	Negative	Pathogens	Contaminants	*P* value
**Fill volume**	**(n= 20,239 BC*)**	**(n= 994 BC*)**	**(n= 329 BC*)**	
Low for age	14,718 (94.6)	604 (3.9)	237 (1.5)	<0.001
Appropriate for age	5,521 (92.0)	390 (6.5)	92 (1.5)	
8 – 10 mL	707 (93.3)	45 (5.9)	6 (0.8)	
≥ 11 mL	248 (92.9)	18 (6.7)	1 (0.4)	
**Type of draw**	**(n = 23,035 BC)**	**(n = 1,146 BC)**	**(n = 400 BC)**	
Peripheral	11,129 (94.4)	374 (3.2)	291 (2.5)	<0.001
Central[%]	11,906 (93.3)	772 (6.0)	109 (0.9)	
Single-lumen CVC	10,762 (93.1)	700 (6.1)	98 (0.8)	0.15
Pooled-lumen CVC	440 (92.2)	36 (7.5)	1 (0.2)	

CVC: central venous catheter
*Blood volume recorded for 21,562/22,139 (97.4%) of all BC bottles
[%]Central lines include arterial and venous catheters

© 2025 Vaugon E et al. *JAMA Network Open*.

eFigure 1. Restrictive blood culture measures enacted via electronic medical records

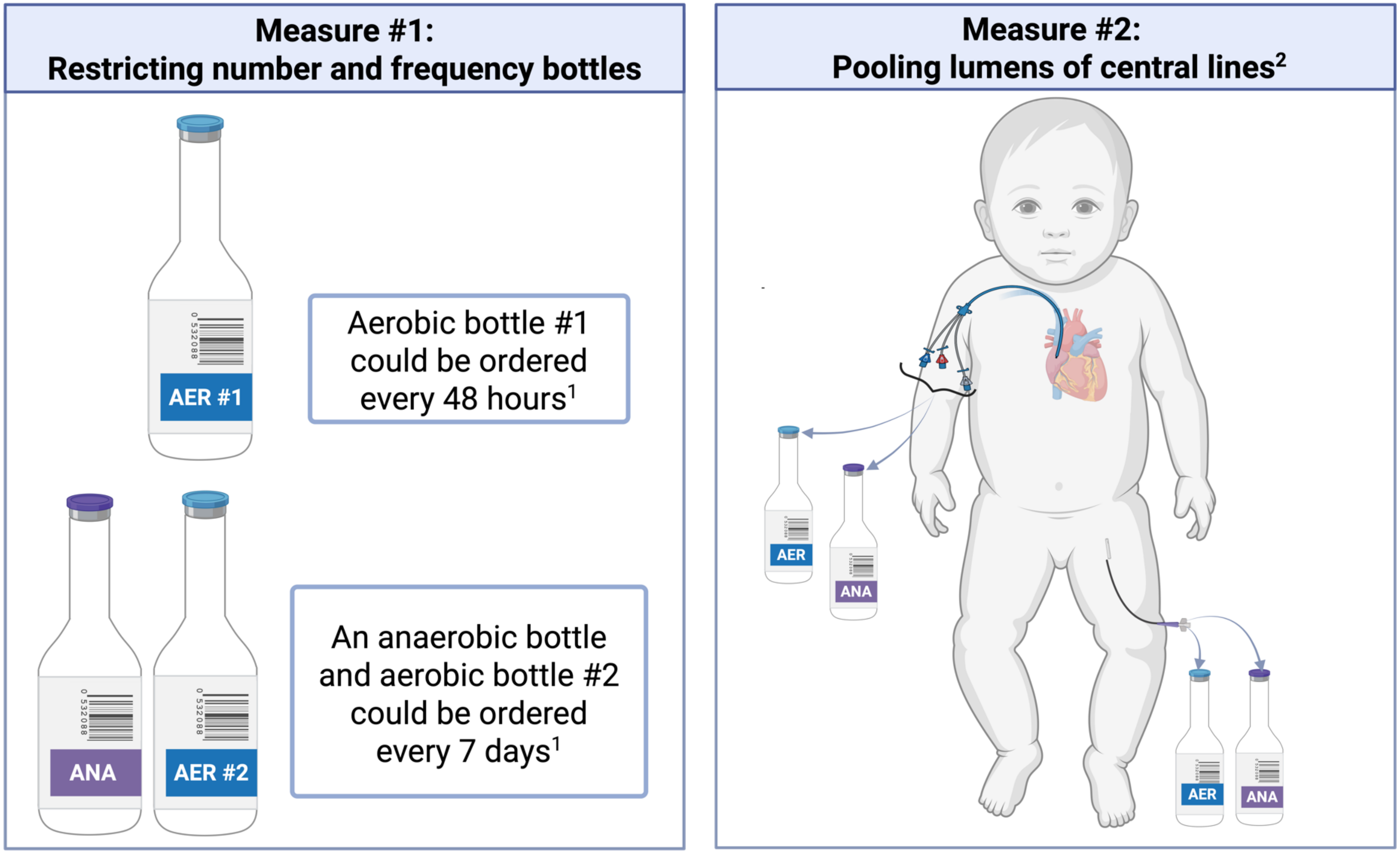

[1]To minimize risks, these restrictions could be overridden at any time with approval from the on-call microbiologist.
[2]For patients with central venous catheters (CVC), all lumens were pooled in the same blood culture bottle. If a patient had more than one CVCs, additional blood culture bottles were provided.
Created in BioRender. Vaugon, E. (2025) https://BioRender.com/kcxmqut.

© 2025 Vaugon E et al. *JAMA Network Open*.

eFigure 2. Syndromic Guidance for Appropriate Blood Culture Use

RECOMMENDATIONS

1. Blood culture **NOT RECOMMENDED** for the following conditions:
   - Isolated fever or leukocytosis in stable patient with no other signs/symptoms of sepsis
   - Stable patient with prosthetic joint infection
   - Non-severe community-acquired pneumonia or hospital-acquired pneumonia
   - Patient with non-severe pneumonia
   - Patient with non-severe cellulitis
   - Lower urinary tract infection (eg. cystitis, prostatitis)
   - Patient with repeatedly negative cultures in the past week
   - All forms of surveillance cultures in patients without suspicion for bloodstream infections
2. Blood culture **RECOMMENDED** for the following conditions:
   - Severe **suspected sepsis** or **septic shock**
   - Neutropenic fever
   - Endocarditis
   - Fever in patient with central venous catheter
   - Meningitis
   - Isolated fever in an infant without identified source
   - Fever with suspected osteomyelitis or septic joint

© 2025 Vaugon E et al. *JAMA Network Open*.

eFigure 3. Classification of contaminants vs pathogens

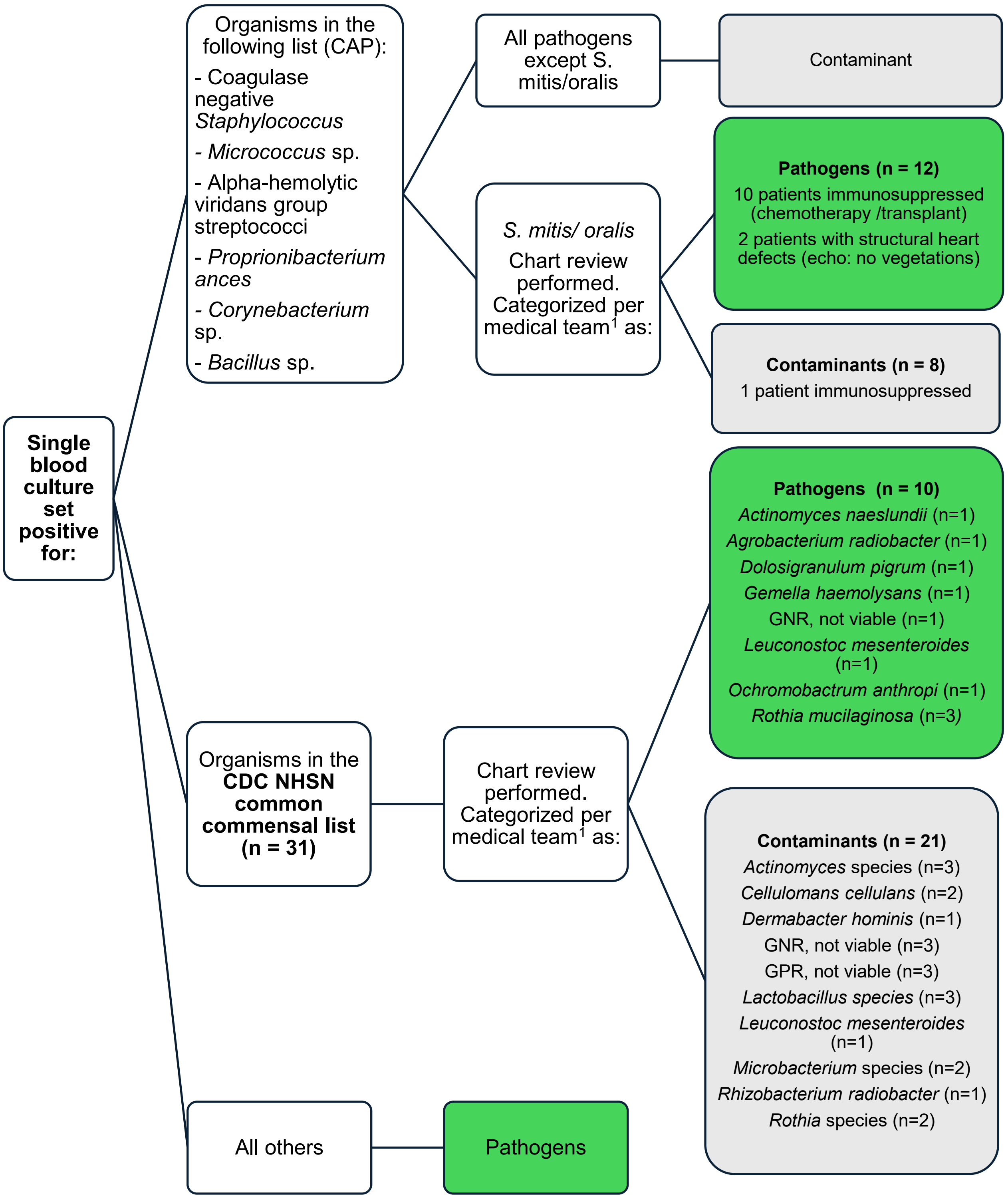

CAP: College of American Pathologists, CDC: Centers for Disease Control and Prevention, NHSN: Healthcare Safety Network

GNR: gram negative rods, GPR: gram positive rod

[1]Chart reviews were conducted to determine whether the identified microbe was considered a contaminant or true pathogen, as recorded in the notes of the primary medical team or infectious diseases consultants

© 2025 Vaugon E et al. *JAMA Network Open*.

eFigure 4. Re-admissions rates, mean length of stay, mortality and mortality secondary to suspected septic shock in the pre- and postintervention period

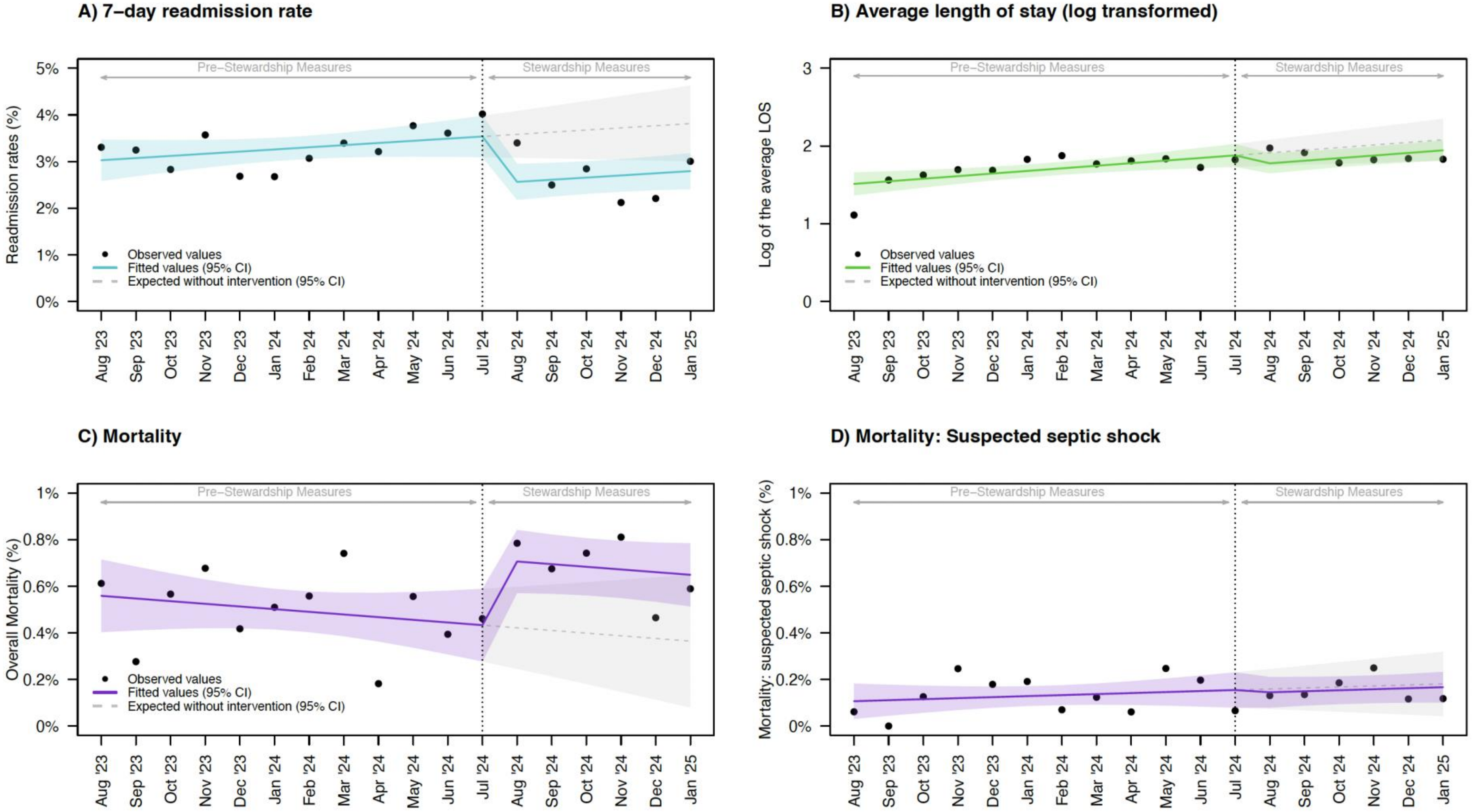

© 2025 Vaugon E et al. *JAMA Network Open*.

eFigure 5. Correlograms and Residuals Analysis

a) Segmented regression model for the monthly blood culture positivity rate in the emergency department

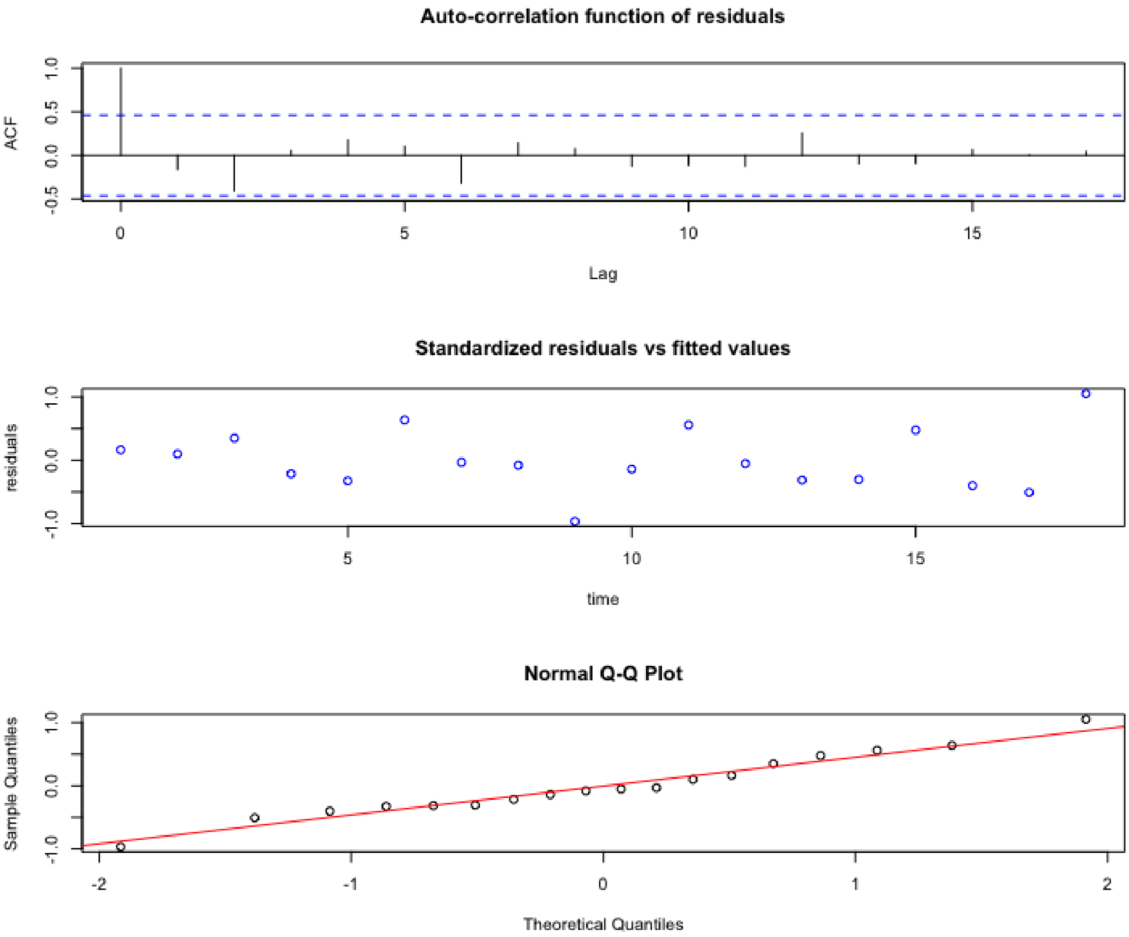

ACF: autocorrelation function

To assess the quality of the segmented linear regression model, we used correlograms (autocorrelation and partial autocorrelation functions which measure the linear relationship between lagged values of a time series) and residuals analysis. Inspection of the correlograms relies on identifying remaining autocorrelation or seasonal pattern of the residuals. The significance of any remaining autocorrelation or seasonality is defined by a correlation higher than +1.96 standard error or lower than -1.96 standard error for each lag of the time series. We checked whether the residuals of the models were normally distributed and had a constant variance over time. The correlograms were satisfactory (no remaining autocorrelation nor seasonal pattern of the residuals).

© 2025 Vaugon E et al. *JAMA Network Open*.

b) Segmented regression model for the monthly blood culture positivity rate in inpatients

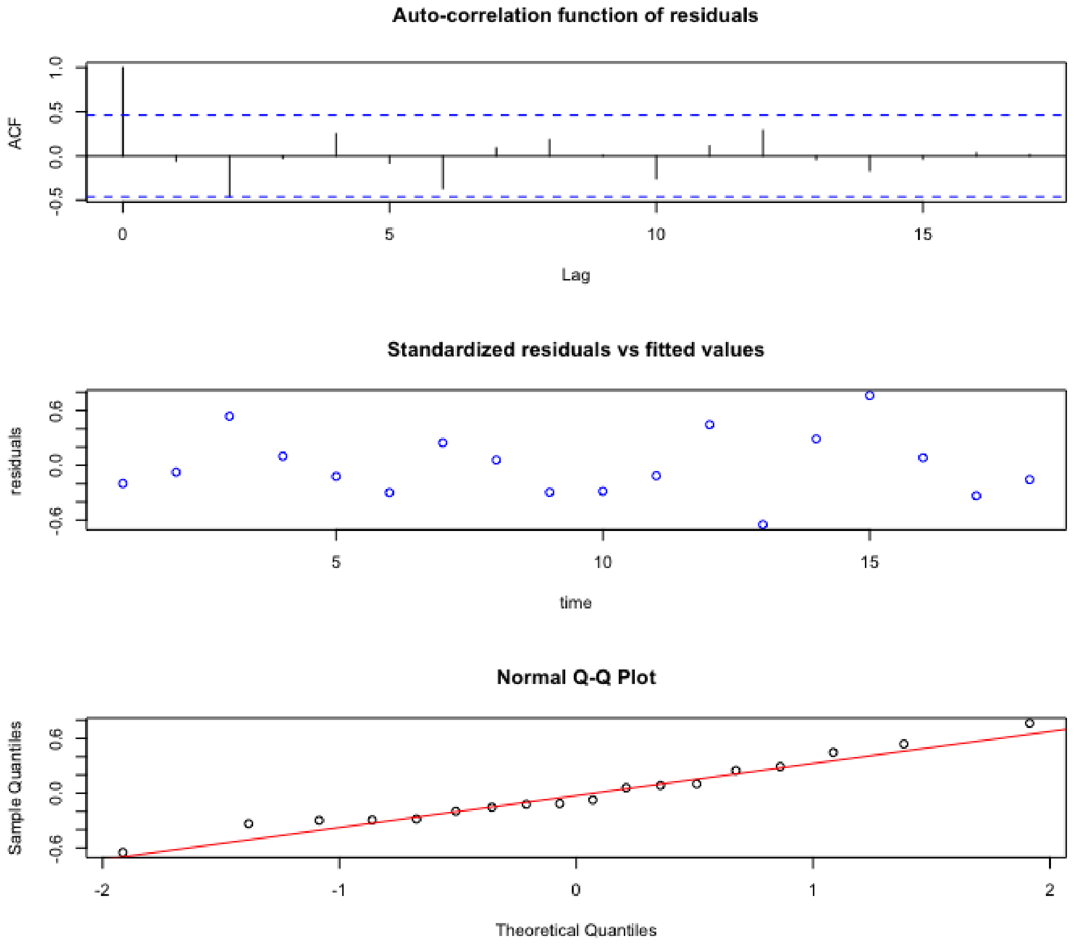

ACF: autocorrelation function

To assess the quality of the segmented linear regression model, we used correlograms (autocorrelation and partial autocorrelation functions which measure the linear relationship between lagged values of a time series) and residuals analysis. Inspection of the correlograms relies on identifying remaining autocorrelation or seasonal pattern of the residuals. The significance of any remaining autocorrelation or seasonality is defined by a correlation higher than +1.96 standard error or lower than -1.96 standard error for each lag of the time series. We checked whether the residuals of the models were normally distributed and had a constant variance over time. The correlograms were satisfactory (no remaining autocorrelation nor seasonal pattern of the residuals).

© 2025 Vaugon E et al. *JAMA Network Open*.

c) Segmented regression model for the monthly blood culture collection rate in the emergency department

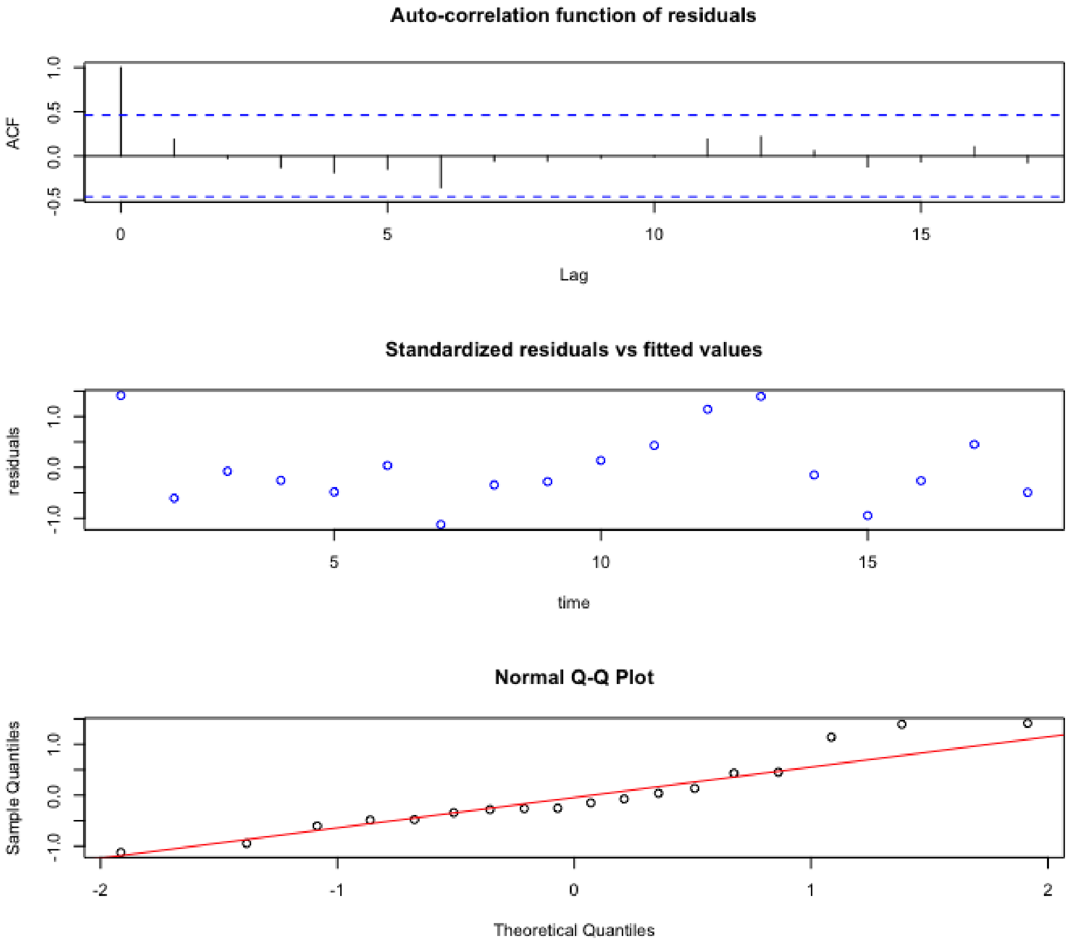

ACF: autocorrelation function

To assess the quality of the segmented linear regression model, we used correlograms (autocorrelation and partial autocorrelation functions which measure the linear relationship between lagged values of a time series) and residuals analysis. Inspection of the correlograms relies on identifying remaining autocorrelation or seasonal pattern of the residuals. The significance of any remaining autocorrelation or seasonality is defined by a correlation higher than +1.96 standard error or lower than -1.96 standard error for each lag of the time series. We checked whether the residuals of the models were normally distributed and had a constant variance over time. The correlograms were satisfactory (no remaining autocorrelation nor seasonal pattern of the residuals).

© 2025 Vaugon E et al. *JAMA Network Open*.

d) Segmented regression model for the monthly blood culture collection rate in inpatients

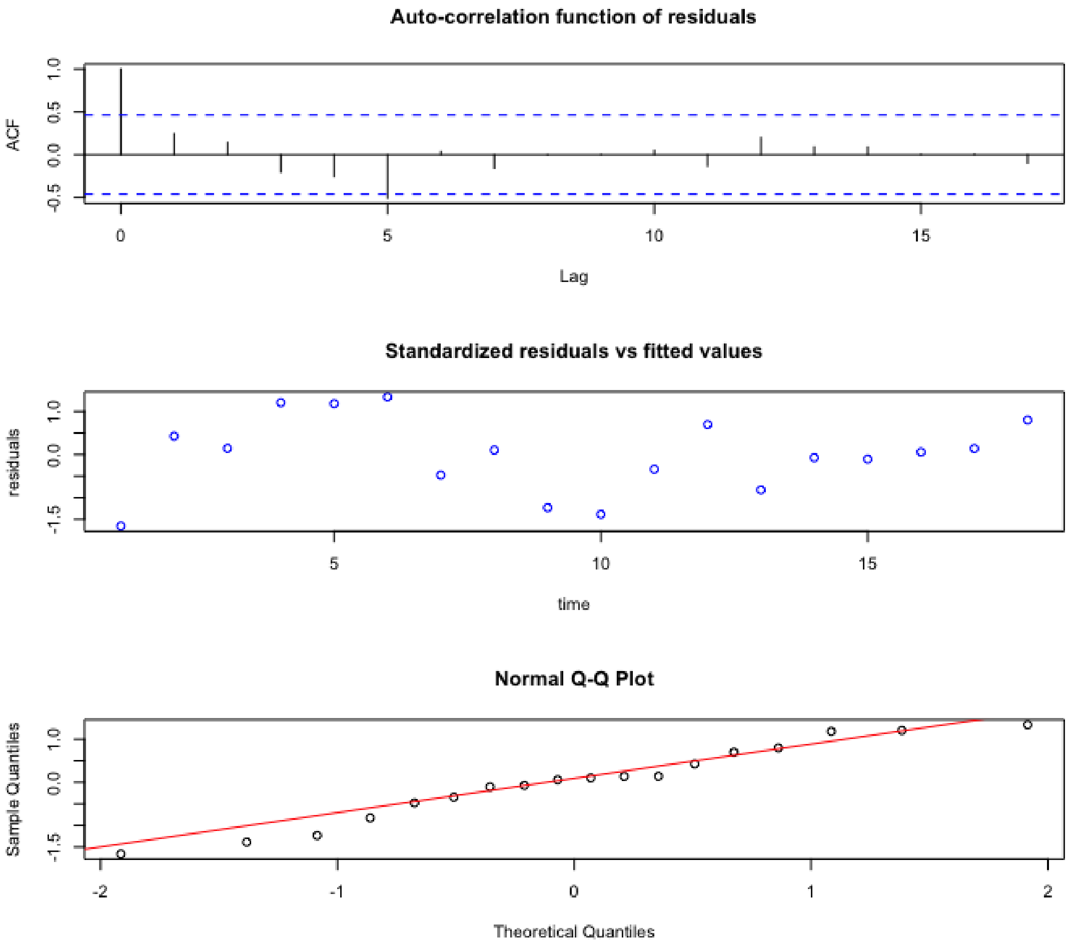

ACF: autocorrelation function

To assess the quality of the segmented linear regression model, we used correlograms (autocorrelation and partial autocorrelation functions which measure the linear relationship between lagged values of a time series) and residuals analysis. Inspection of the correlograms relies on identifying remaining autocorrelation or seasonal pattern of the residuals. The significance of any remaining autocorrelation or seasonality is defined by a correlation higher than +1.96 standard error or lower than -1.96 standard error for each lag of the time series. We checked whether the residuals of the models were normally distributed and had a constant variance over time. The correlograms were satisfactory (no remaining autocorrelation nor seasonal pattern of the residuals).

© 2025 Vaugon E et al. *JAMA Network Open*.

e) Segmented regression model for the readmission rate

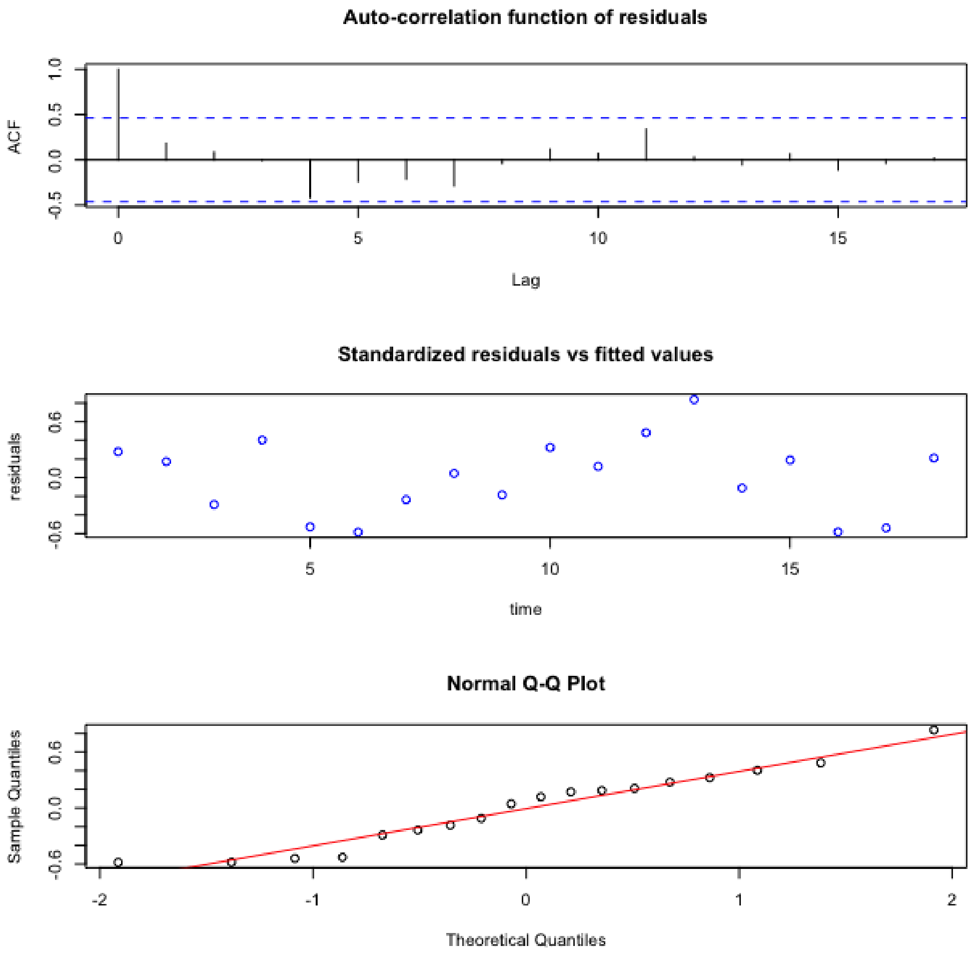

ACF: autocorrelation function

To assess the quality of the segmented linear regression model, we used correlograms (autocorrelation and partial autocorrelation functions which measure the linear relationship between lagged values of a time series) and residuals analysis. Inspection of the correlograms relies on identifying remaining autocorrelation or seasonal pattern of the residuals. The significance of any remaining autocorrelation or seasonality is defined by a correlation higher than +1.96 standard error or lower than -1.96 standard error for each lag of the time series. We checked whether the residuals of the models were normally distributed and had a constant variance over time. The correlograms were satisfactory (no remaining autocorrelation nor seasonal pattern of the residuals).

© 2025 Vaugon E et al. *JAMA Network Open*.

f) Segmented regression model for the length of stay (log-transformed)

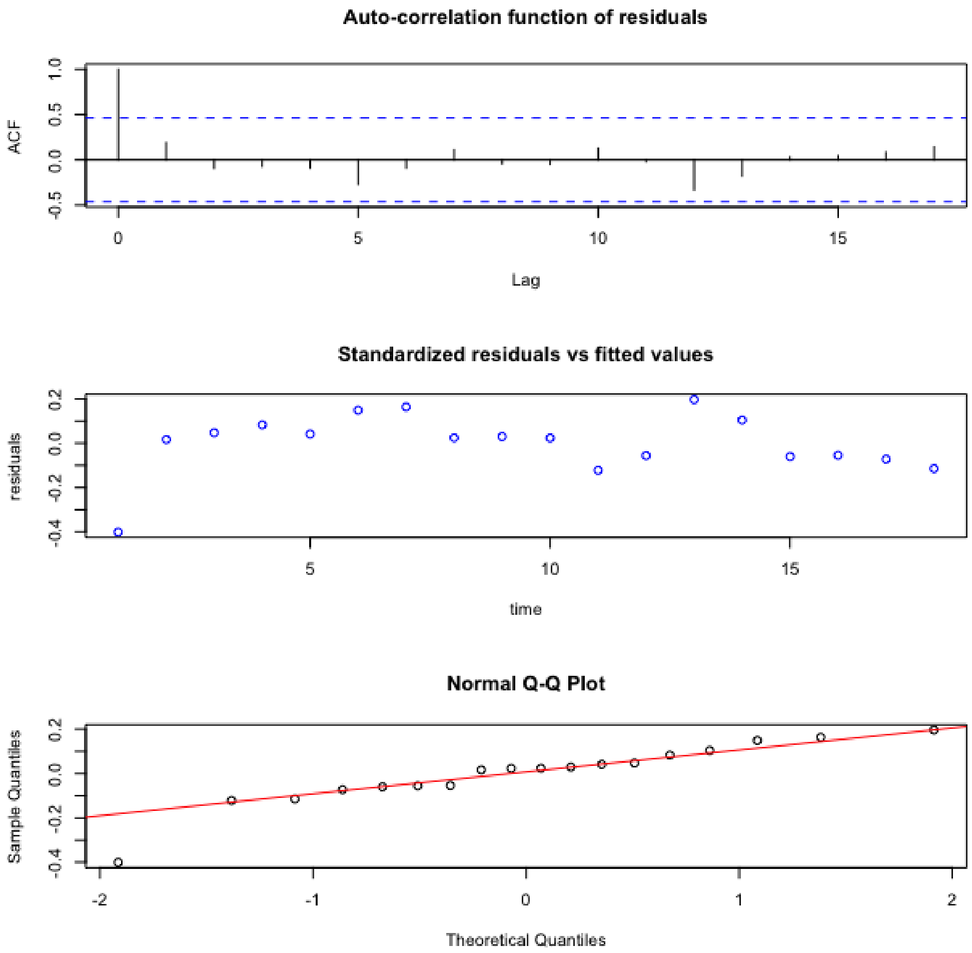

ACF: autocorrelation function

To assess the quality of the segmented linear regression model, we used correlograms (autocorrelation and partial autocorrelation functions which measure the linear relationship between lagged values of a time series) and residuals analysis. Inspection of the correlograms relies on identifying remaining autocorrelation or seasonal pattern of the residuals. The significance of any remaining autocorrelation or seasonality is defined by a correlation higher than +1.96 standard error or lower than -1.96 standard error for each lag of the time series. We checked whether the residuals of the models were normally distributed and had a constant variance over time. The correlograms were satisfactory (no remaining autocorrelation nor seasonal pattern of the residuals).

g) Segmented regression model for the mortality

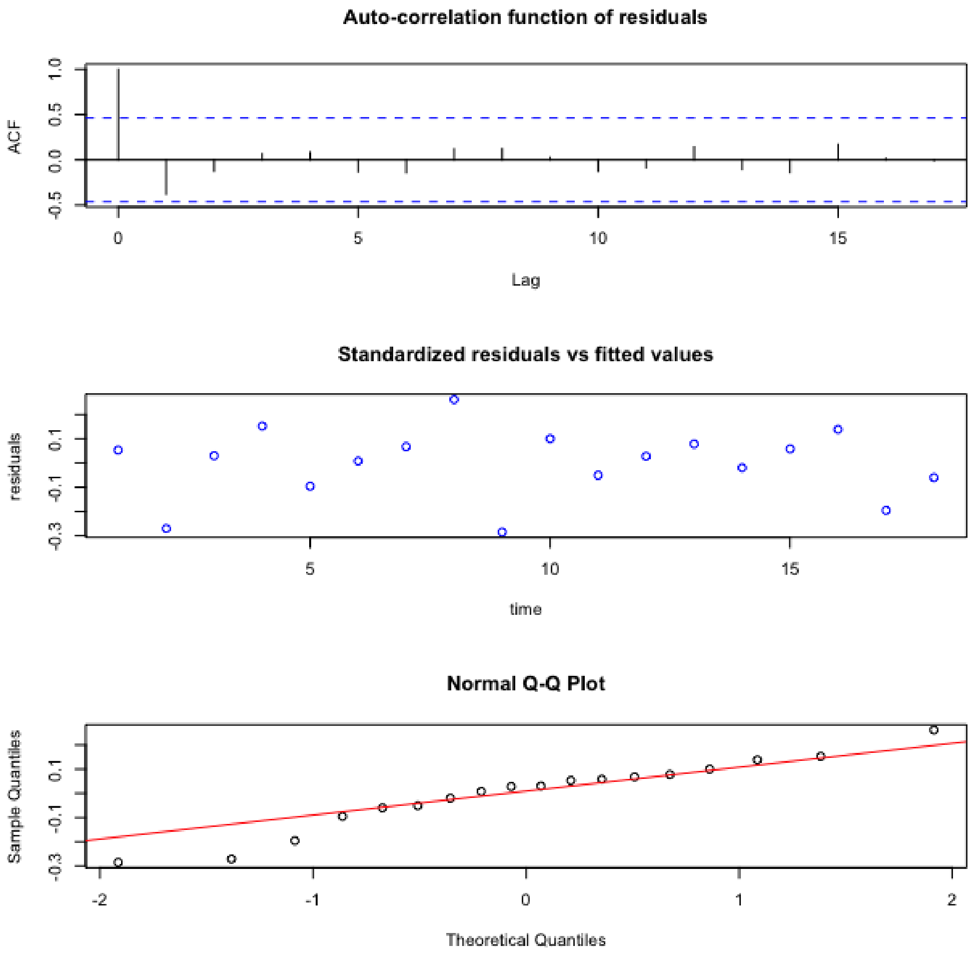

ACF: autocorrelation function

To assess the quality of the segmented linear regression model, we used correlograms (autocorrelation and partial autocorrelation functions which measure the linear relationship between lagged values of a time series) and residuals analysis. Inspection of the correlograms relies on identifying remaining autocorrelation or seasonal pattern of the residuals. The significance of any remaining autocorrelation or seasonality is defined by a correlation higher than +1.96 standard error or lower than -1.96 standard error for each lag of the time series. We checked whether the residuals of the models were normally distributed and had a constant variance over time. The correlograms were satisfactory (no remaining autocorrelation nor seasonal pattern of the residuals).

© 2025 Vaugon E et al. *JAMA Network Open*.

h) Segmented regression model for the mortality secondary to septic shock

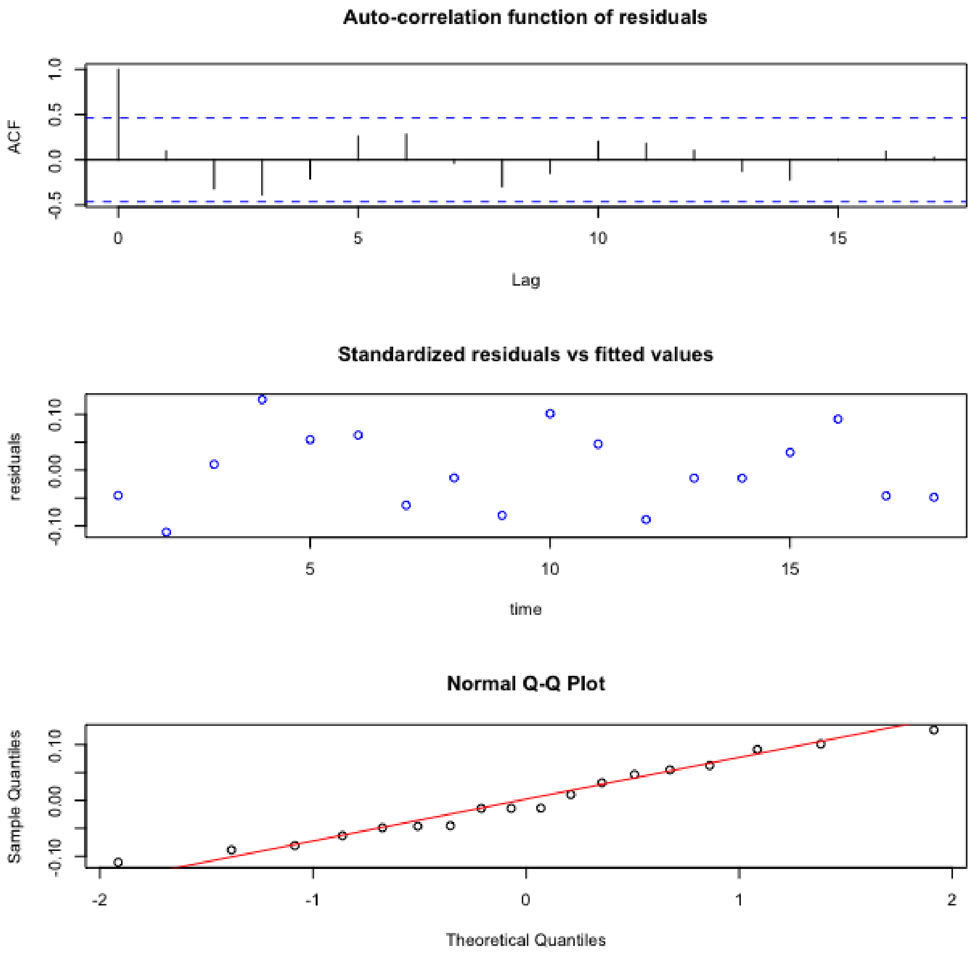

ACF: autocorrelation function

To assess the quality of the segmented linear regression model, we used correlograms (autocorrelation and partial autocorrelation functions which measure the linear relationship between lagged values of a time series) and residuals analysis. Inspection of the correlograms relies on identifying remaining autocorrelation or seasonal pattern of the residuals. The significance of any remaining autocorrelation or seasonality is defined by a correlation higher than +1.96 standard error or lower than -1.96 standard error for each lag of the time series. We checked whether the residuals of the models were normally distributed and had a constant variance over time. The correlograms were satisfactory (no remaining autocorrelation nor seasonal pattern of the residuals).

© 2025 Vaugon E et al. *JAMA Network Open*.
