# Supplementary material for: Rapid Diagnostic Stewardship and Blood Culture Use in a Pediatric Medical Center
Source: JAMA Netw Open. 2025 Oct 6;8(10):e2535580. doi: 10.1001/jamanetworkopen.2025.35580 (PMC12501814; doi:10.1001/jamanetworkopen.2025.35580)
Supplement: Supplement 2. — Data Sharing Statement [file jamanetwopen-e2535580-s002.pdf]

Data Sharing Statement

Vaugon. Rapid Diagnostic Stewardship and Blood Culture Use in a Pediatric Medical Center. *JAMA Netw Open*. Published October 06, 2025. doi:10.1001/jamanetworkopen.2025.35580

Data
Data available: No
